# Developing a multi‐modal MRI radiomics‐based model to predict the long‐term overall survival of patients with hypopharyngeal cancer receiving definitive radiotherapy

**DOI:** 10.1002/wjo2.70001

**Published:** 2025-03-24

**Authors:** Xi‐Wei Zhang, Dilinaer Wusiman, Ye Zhang, Xiao‐Duo Yu, Su‐Sheng Miao, Zhi Wang, Shao‐Yan Liu, Zheng‐Jiang Li, Ying Sun, Jun‐Lin Yi, Chang‐Ming An

**Affiliations:** ^1^ Department of Head and Neck Surgical Oncology National Cancer Center/National Clinical Research Center for Cancer/Cancer Hospital, Chinese Academy of Medical Sciences and Peking Union Medical College Beijing China; ^2^ Purdue Institute for Cancer Research, Purdue University West Lafayette Indiana USA; ^3^ Departments of Radiation Oncology National Cancer Center/National Clinical Research Center for Cancer/Cancer Hospital, Chinese Academy of Medical Sciences and Peking Union Medical College Beijing China; ^4^ Departments of Radiation National Cancer Center/National Clinical Research Center for Cancer/Cancer Hospital, Chinese Academy of Medical Sciences and Peking Union Medical College Beijing China; ^5^ Department of Radiation Oncology Harbin Medical University Cancer Hospital Harbin China; ^6^ Blot Info & Tech (Beijing) Co. Ltd Beijing China; ^7^ State Key Laboratory of Oncology in South China, Collaborative Innovation Center of Cancer Medicine, Guangdong Key Laboratory of Nasopharyngeal Cancer Diagnosis and Therapy Sun Yat‐sen University Cancer Center Guangzhou China

**Keywords:** hypopharyngeal cancer, machine learning, Magnetic Resonance Imaging (MRI), radiomics, survival analysis

## Abstract

**Objective:**

The aim of this study is to develop a multimodal MRI radiomics‐based model for predicting long‐term overall survival in hypopharyngeal cancer patients undergoing definitive radiotherapy.

**Methods:**

We enrolled 207 hypopharyngeal cancer patients who underwent definitive radiotherapy and had 5‐year overall survival outcomes from two major cancer centers in China. Pretreatment MRI images and clinical features were collected. Regions of interest (ROIs) for primary tumors and lymph node metastases (LNM) were delineated on T2 and contrast‐enhanced T1 (CE‐T1) sequences. Principal component analysis (PCA), support vector machine (SVM), and 5‐fold cross‐validation were used to develop and evaluate the models.

**Results:**

Multivariate Cox regression analysis identified age under 50 years, advanced T stage, and N stage as risk factors for overall survival. Predictive models based solely on clinical features (Model A), single radiomics features (Model B), and their combination (Model C) performed poorly, with mean AUC values in the validation set of 0.663, 0.772, and 0.779, respectively. The addition of multimodal LNM and CE‐T1 radiomics features significantly improved prediction accuracy (Models D and E), with AUC values of 0.831 and 0.837 in the validation set.

**Conclusion:**

We developed a well‐discriminating overall survival prediction model based on multimodal MRI radiomics, applicable to patients receiving definitive radiotherapy, which may contribute to personalized treatment strategies.

## INTRODUCTION

Hypopharyngeal cancer (HPC) is a relatively rare malignant tumor, accounting for about 6% of all head and neck malignancies, of which 95% are squamous cell cancers. Due to the lack of warning symptoms, HPC is often diagnosed with local progression or regional lymph node metastasis at first diagnosis. Despite the medical advances in the past few decades, HPC has a relatively worse prognosis than other head and neck malignancies, with an overall 5‐year survival rate of only 33.3%.^1^, ^2^

Due to its high malignancy, the treatment of hypopharyngeal cancer, especially in advanced stages, usually requires multidisciplinary collaboration. In the past few decades, comprehensive treatment strategies based on surgery or definitive radiotherapy have been developed to preserve the laryngeal function without increasing the risk of death. The main treatment methods include surgery‐ and radiotherapy‐based comprehensive treatment modes.^3^ For certain advanced hypopharyngeal cancer patients, the radiotherapy‐based treatment mode, combined with induction chemotherapy, concurrent chemotherapy, or concurrent targeted therapy, can better preserve laryngeal function and improve post‐treatment quality of life without compromising the survival rate, compared to surgery.^4^ However, this preference depends on the clinical TNM staging. For patients with primary stage T4, surgery is strongly recommended due to the poor response to radiotherapy.^5^

Radiomics, a technique that extracts a huge number of image features from different modalities (such as Magnetic Resonance Imaging (MRI), Computed Tomography (CT), Positron Emission Tomography (PET), ultrasound, etc.) and uses machine algorithms to select valuable features and build models to predict tumor‐related outcomes, has been widely studied in clinical applications in recent years. Some studies have used radiomics to perform pre‐treatment predictions and predict recurrence, distant metastasis, and survival for head and neck cancer.^6^, ^7^, ^8^, ^9^

To date, there have been a few radiomics studies in HPC for tumor‐related outcomes or prognosis.^10^, ^11^, ^12^ However, there are some limitations in the current radiomics studies on the prognosis of HPC, such as limited sample size, inconsistent treatment methods, and unsophisticated image processing methods. We collected MRI images from two large cancer centers in China and developed models integrating multi‐modal radiomics and clinical characteristics to improve survival prediction of HPC following definite radiotherapy. This could facilitate personalized treatment planning for patients.

## METHODS

### Patients' enrollment

**Figure 1:**
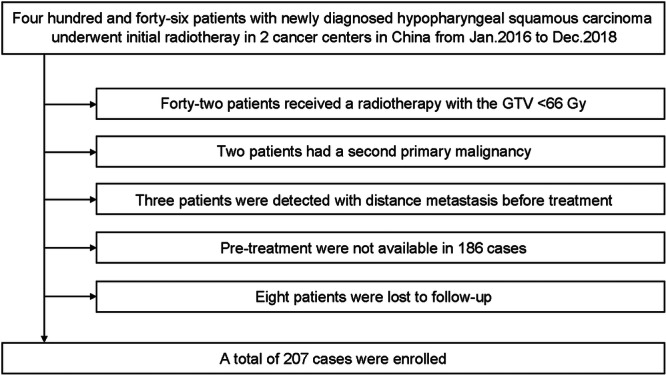
Flowchart of patient selection.

We retrospectively reviewed all patients with pathologically confirmed hypopharyngeal squamous cell cancer who received initial treatment in the radiotherapy departments of two tertiary cancer centers in China, namely Cancer Hospital Chinese Academy of Medical Sciences (CHCAMS) and  Sun Yat‐sen University Cancer Center (SYSUCC), from 2016 to 2018. The exclusion criteria were as follows: (1) patients received radiotherapy dose less than 66 Gy to the primary tumor; (2) patients had a second primary malignancy that might affect the HPC natural course; (3) patients in whom distant metastasis was detected before treatment; (4) patients for whom pre‐treatment MRIs (within one month before radiotherapy or induction chemotherapy) were not available; and (5) patients were lost to follow‐up or had no survival outcome available. The flowchart of patient selection is shown in Figure 1. A total of 207 patients were included, of whom 144 were from CHCAMS and 63 were from SYSUCC.

This study has been registered and approved by the Ethics Committee of National Cancer Center/Cancer Hospital, Chinese Academy of Medical Sciences and Peking Union Medical College. (Approval number: NCC2022C‐682). Considering that it is a retrospective study, signed informed consent was not needed.

### Treatment

All patients received definitive intensity modulated radiation therapy (IMRIT), with gross tumor volume (GTV) more than 66 Gy. The fractionation dose for most patients was 2 Gy, at a fraction of 5 times per week. The target area includes primary and suspected metastatic lymph nodes. The specific regimens of chemotherapy and targeted therapy might vary with time and between centers. Generally, the induced chemotherapy regimens consisted of a combination of taxane‐ and platinum‐based drugs, with or without 5‐FU. Platinum‐based drugs were used in nearly all concurrent chemotherapy. Also, targeted therapy drugs included Cetuximab, Nimotuzumab, and other EGFR‐inhibiting antibodies.

### Data collection

We collected the baseline data, TNM stage, and treatment process of these patients through the Hospital Information System (HIS) of the two hospitals. The baseline data included age, gender, and primary subsite. The relevant treatment information included whether induction chemotherapy, concurrent chemotherapy, or targeted therapy was used. Two senior doctors (one surgeon and one radiologist) restaged all the patients according to both clinical documents and MRI, based on the American Joint Committee on Cancer (AJCC) 8th edition staging system.

The pre‐treatment MRIs of the patients were collected through the PACS system, including the axial images of T1, T2 and contrast‐enhanced T1(CE‐T1) sequences. The patients were required to complete the MRI scanning within 2 weeks before radiotherapy. If the patients underwent induction chemotherapy, the images before chemotherapy should be used. Both centers used 3.0‐T MRI scanners for image acquisition. The imaging sequences included axial and coronal T2‐weighted turbo spin echo (TSE) Dixon sequences, as well as axial, coronal, and sagittal T1‐weighted Dixon sequences.

### Survival outcomes

The patients were followed up for prognosis by two hospitals separately. We performed a preliminary screening by browsing the electronic medical record system of our hospital. We set December 31, 2022 as the deadline. If the patients had outpatient records in the last six months, we used the last outpatient time as the follow‐up endpoint. If the patients had no outpatient records in the last six months, we followed them up by telephone. In the follow‐up, it was determined whether the patients had any form of recurrence, survival status, and possible causes of death. The endpoint of this study was overall survival, which was defined as the time from the initial treatment date to death from any cause. The last follow‐up date was December 2022. Patients who died within 5 years after the initial treatment day were defined as positive events.

### Image processing

To eliminate the potential heterogeneity of images and reduce the influence of scanners, we first performed MRI signal intensity normalization across different centers and machines. The processed images were submitted to a network platform and delineated by an experienced radiation oncologist and an experienced head and neck surgeon. The regions of interest (ROIs) included not only the primary lesions of HPC in each sequence but also all suspicious lymph node metastases (LNM) in the neck. The delineation was finally reviewed by an experienced radiologist.

Radiographically positive lymph nodes were defined by the following criteria: the largest short axis of the retropharyngeal node was ≥5 mm, level II nodes were ≥11 mm, and other nodal levels were ≥10 mm in the axial plane; nodal clusters of three or more (each with a minimum axial dimension of 8‐10 mm); nodes with round shape or central necrosis; and nodes with extranodal extension.^13^

Then, the ROIs of the primary lesions and lymph nodes annotated from the T1, T2, and CE‐T1 sequence were extracted and reconstructed in three dimensions, with the ROI images reconstructed as (1 × 1 × 1)mm voxels. This reconstruction process can reduce the difference in image pixel spacing and form three‐dimensional ROIs.

### Feature extraction

We extracted four types of radiomics features from the three‐dimensional ROIs of different sequences, namely, histogram of oriented gradient (HOG), texture features, wavelet features, and statistical features. The primary lesions and lymph nodes of the T2 sequence and the CE‐T1 sequence each extracted 786 features, including 540 HOG features, 42 texture features, 48 wavelet features, and 156 statistical features. To avoid some patients who only had primary lesions without LNM, we summed up the same features and finally obtained 786 image features for each sequence.

### Feature selection

To select the effective radiomic features, we first removed the invalid features, such as infinite values, null values, or zero‐variance features. Then, we standardized the remaining features to facilitate the dimensionality reduction operation. This study used the principal component analysis (PCA) method to select the most representative features. PCA is a commonly used data dimensionality reduction algorithm, which can project high‐dimensional data into low‐dimensional space, while retaining as much data information as possible. The specific calculation process is shown in Note S1. We ranked the principal components according to their variance contribution ratio, and selected the first 30 principal components as the effective features after dimensionality reduction, which were used to build the model.

### Model development

Based on the 30 radiomic features selected above, we integrated the radiomic labels and the prognosis‐related clinical features, and used the support vector machine (SVM) method to construct an individualized radiomic model. The goal of SVM is to find a hyperplane that maximizes the margin between the closest points of the two classes. By solving the dual problem, the normal vector of the hyperplane can be determined. The specific algorithm of the support vector machine is shown in Note S2.

### Model evaluation

**Figure 2:**
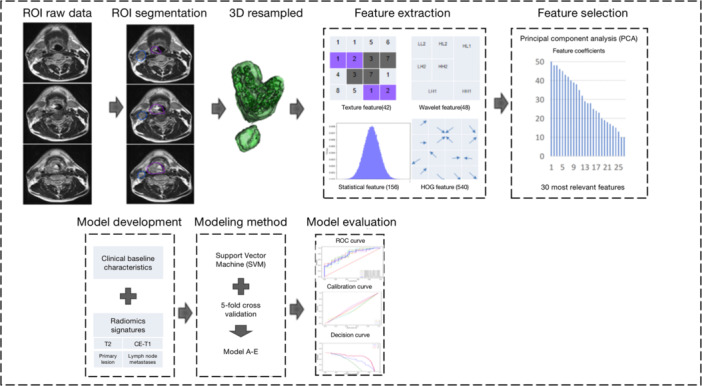
Study workflow. The workflow illustrates the steps of image segmentation, radiomics feature extraction, feature selection, model development, and model evaluation. Image segmentation was performed by experienced radiation oncologists and surgeons, who delineated the regions of interest (ROIs) of the primary lesion and LNM on the T2 and CE‐T1 MRI images. After three‐dimensional reconstruction and resampling of the ROIs, we extracted 786 radiomics features from each sequence of the ROIs and selected the top 30 most relevant features from each sequence by using the principal component analysis (PCA) method. Then, we built multiple prediction models based on different combinations of radiomics features and clinical baseline characteristics using the support vector machine (SVM) method. We applied 5‐fold cross validation to each model, and evaluated their performance using receiver operating characteristic (ROC) curves, calibration curves, and decision curves.

We used three criteria to evaluate the performance of the model: discrimination, calibration, and clinical utility. Discrimination was measured by the area under the receiver operating characteristic curve (AUC), which ranged from 0.50 to 1.0, with values closer to 1.0 indicating stronger discrimination ability of the model. Calibration was assessed by calibration curves, which reflected the consistency between the predicted survival probability and the actual observed survival probability by the model, with closer to the diagonal line indicating better calibration ability of the model. Clinical utility was evaluated by decision curve analysis (DCA), which showed the clinical net benefit of the model at different thresholds, and when the net benefit was greater than 0, it indicated that the model had clinical utility at that threshold. The whole research process is shown in Figure 2.

To split the training and validation sets, we applied the five‐fold cross‐validation method, which randomly shuffled the data of all 207 HPC patients and divided them into five equal‐sized subsets. Then, we repeatedly drew four subsets from these five subsets with replacement as the training set and the remaining one subset as the validation set. In this way, we obtained five different training and validation sets, each subset being exactly the validation set once.

ROI annotation, MRI image standardization and feature extraction, radiomic factor screening, and SVM model training were all performed using Precision Medicine Open Platform 2.0.1 (https://client.blothealth.com). To analyze the high‐risk factors affecting the overall survival of HPC patients, we used univariate and multivariate Cox regression models and performed statistical analysis using SPSS 22.0 software. We considered the results with *P *＜ 0.05 as indicating significant differences. Feature selection and model construction were performed using Pycharm 2021.3.2.

## RESULTS

### The overall outcome and baseline characteristics

This study included 207 patients with HPC, of whom only 2.4% were female, with a median age of 57 ± 10.1 years. Most patients (86%) had primary tumors located in the pyriform sinus, while the postcricoid area had the least primary tumors (2.4%). The distribution of the clinical baseline characteristics of the patients is shown in Table 1. This study excluded patients who were lost to follow‐up, and the median overall follow‐up time was 31 months, with 3‐ and 5‐year overall survival rates of 46.4% and 39.6%, respectively. From Cox univariate and multivariate regression analyses, we found that the age at initial treatment, T stage, and N stage of the patients had a significant negative correlation with overall survival (*P* < 0.05). That is, patients with initial treatment age less than 50 years, T3‐4 stage, and N2‐3 stage had worse overall survival. Different adjuvant treatment modalities (induction chemotherapy, concurrent chemotherapy, or concurrent targeted therapy) showed no significant correlation with overall survival (See Note S3).

**Table 1:** Univariate and multivariate Cox regression analyses of the overall survival of 207 patients with hypopharyngeal cancer based on their baseline characteristics.

Characteristics	*n* (%)	Univariate analysis		Multivariate analysis	
		*HR* (95% *CI*)	*P* value	*HR* (95% *CI*)	*P* value
Age (years)					
<50	57 (27.5)		0.003		0.004
≥50, and<60	74 (35.7)	0.469 (0.303–0.727)	0.001	0.481 (0.311–0.746)	0.001
≥60	76 (36.7)	0.638 (0.421–0.966)	0.034	0.645 (0.425–0.979)	0.039
Gender					
Female	5 (2.4)	1.050 (0.224–3.302)	0.933		
Male	202 (97.6)				
Primary subsite					
Piriform sinus	176 (85.0)		0.348		
Posterior wall	26 (12.6)	1.220 (0.748–1.991)	0.426		
Post‐cricoid	5 (2.4)	1.915 (0.704–5.208)	0.203		
T‐stage					
T1‐2	83 (40.1)	1.449 (1.009–2.082)	0.045	1.507 (1.049–2.167)	0.027
T3‐4	124 (59.9)				
N‐stage					
N0‐1	60 (29.0)	2.039 (1.324–3.140)	0.001	2.044 (1.326–3.150)	0.001
N2‐3	147 (71.0)				

### Selection of radiomics features and model development

Among the extracted radiomic features, 66 were identified as invalid, including 9 with infinite values, 22 with null values, and 35 with zero variance; these invalid features were excluded. The remaining 720 features were screened using Principal Component Analysis (PCA), resulting in the selection of 30 radiomic features with the highest correlation to survival status.

To build a model for predicting the overall survival of HPC patients, we developed 5 models based on different combinations of data sets. Model A only included pure clinical data, Model B only included radiomics features of the primary tumor from the T2 sequence, Model C included both clinical data and radiomics features of the primary tumor from the T2 sequence, Model D added radiomics features of the LNM from the T2 sequence to model C, and model E added radiomics features of both the primary tumor and the LNM from the CE‐T1 sequence to model D.

### Evaluation and comparison of the models

**Figure 3:**
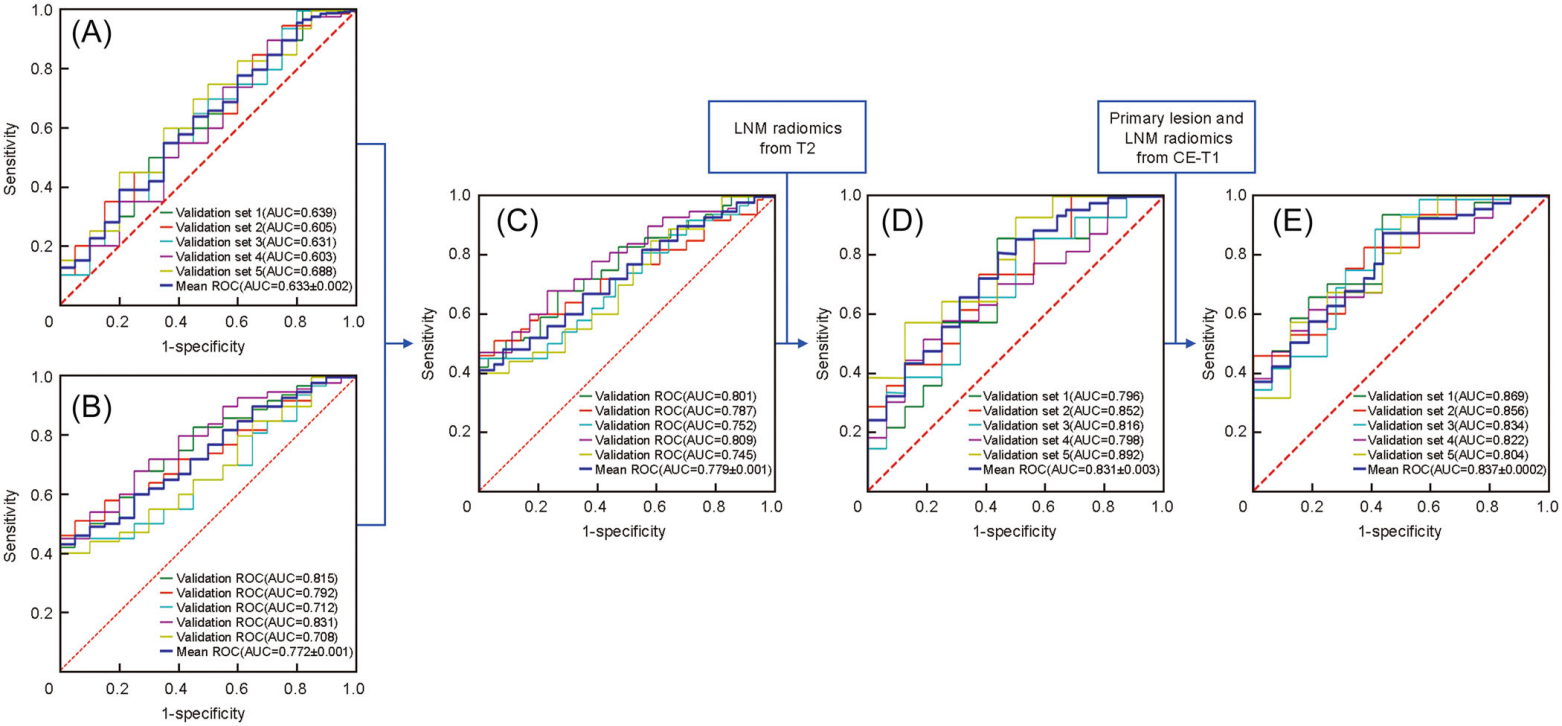
ROC curves of the validation groups for models A‐E using 5‐fold cross‐validation. (A) Model A (pure clinical characteristics), with an average AUC of 0.633 ± 0.002. (B) Model B (primary lesion radiomics from T2), with an average AUC of 0.772 ± 0.001. (C) Model C (clinical characteristics+ primary lesion radiomics from T2), with an average AUC of 0.779 ± 0.001. (D) Model D (clinical characteristics + T2 primary lesion and LNM), with an average AUC of 0.831 ± 0.003. (E) Model E (clinical + T2, CE‐T1 primary lesion, and LNM), with an average AUC of 0.837 + 0.0002.

After using the 5‐fold cross‐validation method, we obtained the following results for the five models. Model A used pure clinical baseline characteristics (age, T‐stage, N‐stage), and had an average AUC of 0.633 ± 0.002 in the validation group. Model B used pure radiomics data from T2 primary lesion images, and had an average AUC of 0.772 ± 0.001 in the validation group (see Table 2 for details of each group). Model C combined the data from Models A and B, and had an average AUC of 0.779 ± 0.001 in the validation group. Model D added radiomics data from T2 LNM to Model C, and had an average AUC of 0.831 ± 0.003 in the validation group. Model E added CE‐T1 enhanced radiomics of primary and metastatic lesions to Model D, and had an average AUC of 0.837 ± 0.0002 in the validation group. The ROC curves of the validation groups for each model are shown in Figure 3.

**Table 2:** Performance of Models A to E.

Group	Model	ACC	SE	SP	PPV	NPV	AUC
Training group	A	0.901	0.851	0.929	0.870	0.918	0.943
	B	0.917	0.896	0.929	0.878	0.940	0.968
	C	0.924	0.894	0.940	0.894	0.940	0.973
	D	0.932	0.896	0.952	0.915	0.941	0.990
	E	0.954	0.957	0.952	0.918	0.976	0.993
Validation group	A	0.719	0.636	0.762	0.583	0.800	0.663
	B	0.750	0.636	0.810	0.636	0.810	0.772
	C	0.781	0.727	0.810	0.667	0.850	0.779
	D	0.813	0.727	0.857	0.727	0.857	0.831
	E	0.844	0.818	0.857	0.750	0.900	0.837

Abbreviations: ACC, Accuracy, AUC, Area under the curve; NPV, Negative predictive value; PPV, Positive predictive value; SE, Sensitivity; SP, Specificity.

**Figure 4:**
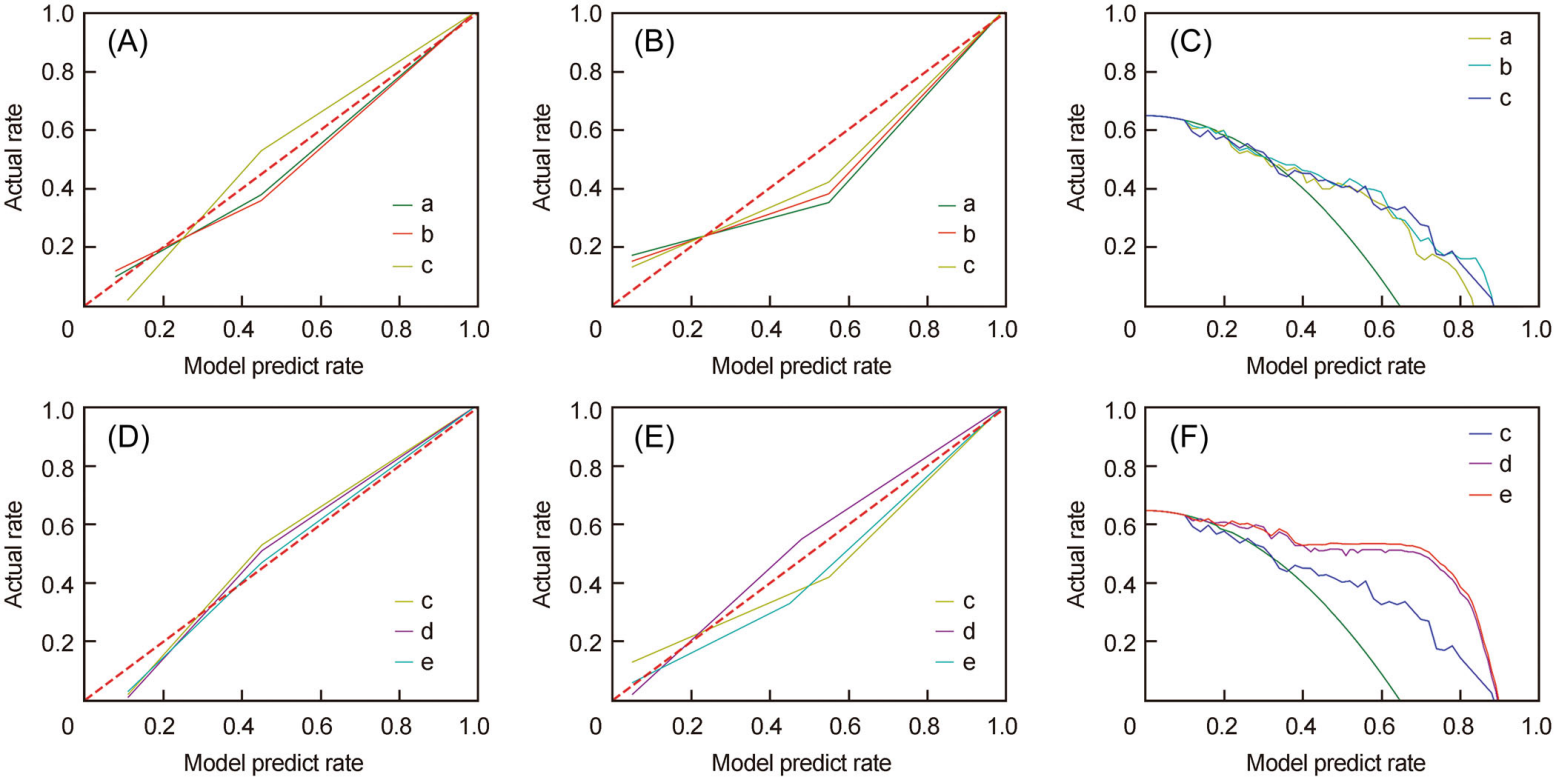
Calibration curves and decision curves for the five prediction models. (A–C), respectively, show the calibration curves of training and validation sets, and decision curves for Models A, B, and C. (D–F) show the calibration curves of training and validation sets, and decision curves for Models D, E, and F, respectively. The diagonal dashed red line in calibration curves represents the perfect performance of an ideal model. A better prediction performance is indicated by a closer fit to the diagonal dashed red line. The green line in decision curves represents the scenario that all patients survived after 5‐year follow‐up, whereas the gray line represents the scenario that no patients survived.

Model A vs. B vs. C (Figure 4A,B) and Model C vs. D vs. E (Figure 4D,E) demonstrated that the radiomics‐based prediction models showed good agreement between predicted and actual 5‐year overall survival both in the training and validation sets. The decision curve analyses for the A vs. B vs. C models and the C vs. D vs. E models are presented in Figure 4C,F. The decision curves showed that if the threshold probability ranged from 36% to 89% for Model C, it could add more benefit (a net benefit could be derived) than a treat‐all or treat‐none scheme; if the threshold probability ranged from 17% to 91%, Model E added more benefit than a treat‐all or treat‐none scheme.

## DISCUSSION

HPC is a rare but highly malignant head and neck tumor. For patients with newly diagnosed hypopharyngeal squamous cell cancer, accurate prediction of long‐term survival outcomes before treatment can not only guide individualized treatment plans but also improve patient compliance and satisfaction. Traditional prognostic analysis mainly relies on clinical and pathological factors, but these factors are difficult to quantify and apply to clinical prediction. In recent years, with the advancement of artificial intelligence technology, radiomics has shown advantages in prognostic prediction of various tumors. However, there are few radiomics studies on HPC, and there are some limitations, such as inconsistent treatment methods and unsophisticated image processing methods.

To overcome these problems, we used data from two major cancer centers in northern and southern China, selecting patients who received definitive radiotherapy. We included T2 radiomics of the primary lesion MRI, as well as LNM and CE‐T1 multi‐modal radiomics, to develop a series of survival prediction models for HPC. The prediction performance of the models based on only clinical or single radiomics features (models A, B, and C) was poor. The incorporation of multi‐modal LNM and CE‐T1 radiomics features (models D and E) substantially enhanced the prediction accuracy, resulting in higher mean AUC values of 0.831 and 0.837 in the validation set.

Hypopharyngeal cancer has a relatively poor prognosis, and several risk factors for adverse outcomes have been identified by previous studies, including higher clinical stage, older age, male gender, and poor performance scores.^14^ Moreover, shorter survival times have been found for black patients and those of lower socioeconomic status.^15^ In our study, it was found that worse overall survival rates were associated with initial treatment age ＜50 y old, T3‐4 stage, and N2‐3 stage. Surprisingly, this phenomenon of the worst prognosis for patients younger than 50 years has not been reported before. We speculate that, compared to older patients, younger patients with HPC may have different etiological factors that result in more aggressive biological behavior of the tumor.

To predict the treatment outcome of HPC patients, some studies have developed predictive models using machine learning. For example, Heng et al.^16^ developed a model based on the clinical data of 198 HPC patients who underwent surgery only without postoperative adjuvant treatment, and achieved a discrimination with C‐index = 0.767 in the validation set. Mo et al.^17^ developed a model to predict progression‐free survival (PFS) based on the clinical data and CT radiomics features of 113 HPC patients who received chemoradiotherapy, using the least absolute shrinkage and selection operator (LASSO) methods, and obtained a C‐index of 0.756 in the validation set.

MRI has been widely used in radiomics research, as it can provide better differentiation between tumor and normal tissue, and more diverse radiomic features by using various sequences and parameters, compared with CT. We applied most of the conventional radiomic methods and found that Model C, which combined clinical data and single‐modal radiomics (T2 primary lesion), did not significantly improve the discrimination, with an AUC of only 0.779. This value was similar to the prediction ability reported by Chen et al.^18^ They included 197 patients from two centers who received radiotherapy or surgery for HPC and developed a model based on CE‐T1 sequence primary lesion radiomics, which had a c‐index of 0.75 in the validation set. In another study, Siow et al.^19^ included 198 HPC patients who received concurrent chemoradiotherapy and used CE‐T1 sequence primary tumor radiomics. Their final OS and PFS model, which combined radiomic‐clinical data, had an AUC of only 0.671 and 0.675, respectively.

From the above study of clinical risk characteristics, we can see that the most significant factor in the multivariate analysis was high N‐stage, and the mortality risk of N2‐3 cases was about 2 times that of N0‐1 patients (*HR* = 2.004). Therefore, we believe that it is not enough to fully predict the prognosis by only adding radiomics of primary lesions in radiomics research. As expected, after adding radiomics of LNMs, Model D showed a significant improvement in prediction power compared with Model C, and the AUC of the validation group increased to 0.831.

However, when we further added CE‐T1 sequence radiomics, the prediction power did not improve significantly, with only a 0.006 increase in AUC, and Model E's decision curve almost overlapped with Model D's. This indicates that simply adding CE‐T1 sequence radiomics could not effectively improve the model performance. Therefore, we speculate that the contribution of different MRI sequences of radiomics to the model prediction ability may be less than that of adding LNM radiomics.

## CONCLUSION

We developed a series of models based on clinical and multi‐modal radiomics data from HPC patients, who received definitive radiotherapy, of two major cancer centers in China. The performance of conventional radiomics model based on clinical or single‐radiomics features was poor, while the incorporation of multi‐modal LNM and CE‐T1 radiomics features substantially enhanced the prediction accuracy, resulting in higher AUC values of 0.831 and 0.837 in the validation set.

## AUTHOR CONTRIBUTIONS

**Xi‐Wei Zhang**: Statistical analysis (equal); manuscript preparation (lead); manuscript editing (lead). **Dilinaer Wusiman**: Manuscript preparation (support); manuscript editing (support). **Ye Zhang**: Data analysis and interpretation (equal). **Xiao‐Duo Yu**: Data analysis and interpretation (equal). **Su‐Sheng Miao**: Data acquisition (equal). **Zhi Wang**: Statistical analysis (equal). **Shao‐Yan Liu**: Manuscript review (equal). **Zheng‐Jiang Li**: Manuscript review (equal). **Ying Sun**: Data acquisition (equal). **Jun‐Lin Yi**: Manuscript review (equal). **Chang‐Ming An**: Study concepts (lead); study design (lead).

## CONFLICT OF INTEREST STATEMENT

The authors declare no conflicts of interest.

## ETHICS STATEMENT

This study has been registered and approved by the Ethics Committee of National Cancer Center/Cancer Hospital, Chinese Academy of Medical Sciences and Peking Union Medical College. (Approval number: NCC2022C‐682). Considering that this is a retrospective study, informed consent was not needed.

## Supporting information

Supporting Information.

Supporting Information.

Supporting Information.

## Data Availability

Data associated with this study are available upon reasonable request to the corresponding author.
