# Supplementary material for: Developing a multi‐modal MRI radiomics‐based model to predict the long‐term overall survival of patients with hypopharyngeal cancer receiving definitive radiotherapy
Source: World J Otorhinolaryngol Head Neck Surg. 2025 Mar 24;11(3):440–8. doi: 10.1002/wjo2.70001 (PMC12418344; doi:10.1002/wjo2.70001)
Supplement: Supplementary file 1 — Supporting Information. [file WJO2-11-440-s003.docx]

**Supplementary Note 1.**

Principal component analysis (PCA) is a classic data dimensionality reduction algorithm, which aims to transform the original n-dimensional features into k-dimensional new features through orthogonal transformation. These new features are also called principal components, which can retain the maximum amount of information from the original data.

The specific steps of PCA algorithm are as follows:

1. Perform mean normalization for each feature, that is, subtract the mean value of that feature, so that its mean value is zero.
2. Calculate the covariance matrix of the data after mean normalization.
3. Use singular value decomposition (SVD) method to solve the eigenvalues and eigenvectors of the covariance matrix.
4. Sort the eigenvalues in descending order, and select the largest 30 eigenvalues and their corresponding 30 eigenvectors. Form a feature vector matrix with these 30 eigenvectors as column vectors.
5. Multiply the original data by the feature vector matrix to obtain the transformed data, which are the principal components.
6. Rank the principal components according to their variance contribution ratio, and select the first 30 principal components as the effective features after dimensionality reduction, which are used to build the model.
