# Supplementary material for: Developing a multi‐modal MRI radiomics‐based model to predict the long‐term overall survival of patients with hypopharyngeal cancer receiving definitive radiotherapy
Source: World J Otorhinolaryngol Head Neck Surg. 2025 Mar 24;11(3):440–8. doi: 10.1002/wjo2.70001 (PMC12418344; doi:10.1002/wjo2.70001)
Supplement: Supplementary file 2 — Supporting Information. [file WJO2-11-440-s002.docx]

**Supplementary Note 2.**

The objective of support vector machine (SVM) is to find a hyperplane that separates the two classes, and maximizes the distance between the hyperplane and the closest points of the two classes. By doing so, the normal vector of the hyperplane can be determined.

SVM algorithm process:

1. Use the training set to construct the feature X-label y model: Suppose the training set data is: *D_1._*


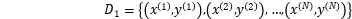


There are N samples, each sample x (vector) has n features; y is the category to which x belongs, assuming there are k categories.

(2) Find a hyperplane that divides the data set into two classes, and maximizes the margin between the closest sample points of the two classes.
