# Supplementary material for: Developing a multi‐modal MRI radiomics‐based model to predict the long‐term overall survival of patients with hypopharyngeal cancer receiving definitive radiotherapy
Source: World J Otorhinolaryngol Head Neck Surg. 2025 Mar 24;11(3):440–8. doi: 10.1002/wjo2.70001 (PMC12418344; doi:10.1002/wjo2.70001)
Supplement: Supplementary file 3 — Supporting Information. [file WJO2-11-440-s001.docx]

Table. Univariate and multivariate cox regression analyses of the overall survival of 207 patients with hypopharyngeal cancer based on treatment regimens.

| Characteristics | n(%) | Univariate Analysis | | Multivariate Analysis | |
| --- | --- | --- | --- | --- | --- |
|  |  | HR（95% CI） | P | HR(95% CI) | P |
| Induced chemotherapy |  |  |  |  |  |
| No | 149(72.0) | 1.066(0.871-1.305) | 0.535 | / | / |
| Yes | 58(28.0) |  |  |  |  |
| Concurrent chemoradiotherapy |  |  |  |  |  |
| No | 74(35.7) | 0.990(0.827-1.186) | 0.916 | / | / |
| Yes | 133(64.3) |  |  |  |  |
| Concurrent Targeted therapy |  |  |  |  |  |
| No | 167(80.7) | 1.001(0.801-1.249) | 0.996 | / | / |
| Yes | 40(19.3) |  |  |  |  |
